# Clinical features of Kawasaki disease initially mimicking retropharyngeal abscess: a retrospective analysis

**DOI:** 10.1186/s12969-022-00778-4

**Published:** 2022-12-13

**Authors:** Yong-chao Chen, Hong-guang Pan, De-sheng Jia, Hao-cheng Wang, Lan Li, Yi-shu Teng

**Affiliations:** 1 Department of Otorhinolaryngology, Shenzhen Children’s Hospital, 7019 Yitian Road, Futian District, Shenzhen, Guangdong China; 2 Department of Otorhinolaryngology, Shenzhen Children’s Hospital, China Medical University, Shenzhen, Guangdong China

**Keywords:** Children, Kawasaki disease, Retropharyngeal abnormality, Retropharyngeal abscess, Retropharyngeal cellulitis

## Abstract

**Objective:**

Incomplete Kawasaki disease (IKD) initially presenting as retropharyngeal abnormality is very rare and is prone to misdiagnosis and missed diagnosis, often leading to poor prognosis. Most patients were misdiagnosed with retropharyngeal abscesses. Here, we describe and compare IKD patients initially presenting with retropharyngeal abnormalities, typical KD patients without retropharyngeal abnormalities and retropharyngeal abscess patients.

**Methods:**

We performed a retrospective case–control study comparing IKD patients initially presenting with retropharyngeal abnormalities to both KD patients without retropharyngeal abnormalities and retropharyngeal abscess patients admitted to Shenzhen Children’s Hospital between January 2016 and December 2021.

**Results:**

We evaluated data from 10 IKD patients initially presenting with retropharyngeal abnormalities (Group A), 20 typical KD patients (Group B) and 16 surgical drainage confirmed retropharyngeal abscess patients (Group C). Compared to Group B, we observed that Group A was older and had a more intense inflammatory response. On the day of admission, Groups A and C had similar early clinical presentations, and there were no significant differences in any major signs or symptoms. Close observation for the development of new KD signs and symptoms and unresponsiveness to empirical antibiotic therapy after 3 days is extremely important. The CRP (*p* = 0.011), AST (*p* = 0.002) and ALT (*p* = 0.013) levels were significantly higher and the WBC (*P* = 0.040) levels were significantly lower in Group A than in Group C. Neck radiological findings, such as the presence of ring enhancement (*p* = 0.001) and mass effects on the airway, are also useful tools for distinguishing these two diseases.

**Conclusion:**

The careful observation of the signs and symptoms of this disease and the comprehensive analysis of the laboratory tests and neck radiological findings may help clinicians become aware of retropharyngeal abnormality as an atypical presentation of KD. Then, unnecessary treatments could be reduced, and the occurrence of serious complications can be avoided.

## Introduction

Kawasaki disease (KD), which was previously called cutaneous mucosal lymph node syndrome, is an acute, self-limiting vasculitis that predominantly affects children less than 5 years of age. KD mainly involves the middle and small arteries, especially the coronary artery, and can cause a serious incidence of cardiovascular complications, including coronary artery aneurysms (CAAs) and coronary artery stenosis or thrombosis [1]. KD has had increasing incidence worldwide, with the highest rates seen among Asian/Pacific Islanders at 29.8 per 100,000 children < 5 years. KD has various clinical presentations, and typical clinical manifestations include the presence of a fever lasting five or more days, bilateral conjunctival injection, oral changes such as cracked and erythaematous lips and strawberry tongue, cervical lymphadenopathy, extremity changes such as erythema or palm and sole desquamation, and polymorphous rash [1, 2]. Its diagnosis mainly relies on clinical manifestations due to the lack of reliable objective indicators. It is not difficult to diagnose KDs when they have a typical clinical manifestation. However, the diagnosis of incomplete KD (IKD) can be difficult. Some IKD cases initially presented as retropharyngeal abnormalitys, without the additional clinical criteria for the diagnosis of classic KD. They are very rare and are prone to misdiagnosis and missed diagnosis, often leading to poor prognosis. Most patients were misdiagnosed as retropharyngeal abscess because retropharyngeal abscess also mainly affects children, causing fever, cervical lymphadenopathy, leukocytosis, and elevated serum C-reactive protein (CRP) [3, 4]. Because the treatments of the two diseases are quite different and the diseases can be fatal if an appropriate treatment is not provided, distinguishing them is important. However, only a few case reports or case series have been reported in the literature [5–12], and very few retrospective case–control studies have been conducted in this regard. Here, our retrospective study afforded the unique opportunity to describe and compare IKD patients initially presenting with retropharyngeal abnormalities, typical KD patients without retropharyngeal abnormalities and retropharyngeal abscess patients from a single centre. We sought to identify clinical, laboratory, and imaging features that distinguish patients with IKD initially presenting as retropharyngeal abnormalitys and retropharyngeal abscesss. To increase knowledge and awareness of this intriguing disease and to avoid delays in treatment and unnecessary surgery in KD children initially presenting with retropharyngeal abnormalities.

## Patients and methods

### Patients

All procedures performed in this study involving human participants were in accordance with the 1964 Declaration of Helsinki and its later amendments or comparable ethical standards. This study employed retrospective medical record reviews of children who were admitted with discharge diagnoses suggestive of KD or retropharyngeal abscess in Shenzhen Children’s Hospital between January 2016 and December 2021. There were 1516 children with discharge diagnoses of KD and 123 children with discharge diagnoses of retropharyngeal abscess in the Inpatient Discharge Registry. Ultimately, we included 10 IKD patients initially presenting as retropharyngeal abnormality (group A),20 KD patients without retropharyngeal abnormality (group B) and 16 surgical drainage confirmed retropharyngeal abscess patients (group C). In 1516 KD patients, 10 patients (0.66%) initially presented as retropharyngeal abnormality (group A), and they were suspected as having retropharyngeal abscess from their clinical findings accompanied by retropharyngeal low-density areas on CT, and were admitted or transferred to the pediatric ENT department. They all were finally diagnosed with IKD according to the American Heart Association diagnosis criteria in 2017 [1]. In addition, for each KD case initially presenting as retropharyngeal abnormality, two control children were randomly selected from KD without retropharyngeal abnormality children (Group B). And 16 patients (13.01%) confirmed by incision and drainage of the abscess (group C) of the 123 retropharyngeal abscess patients. Immunocompromised children, traumatized children, and those with cervical structural anomalies were excluded. The neck CT showing peritonsillar abscess, superficial cellulitis, or superficial lymph node abscess without accompanying retropharyngeal inflammation were also excluded. In group A and group C, they all presented with fever and cervical lymphadenopathy at an early phase of the disease (around the day of admission), so neck CT or neck MRI was performed on them to rule out deep neck infection.

### Data collection

The enrolled children’s clinical histories were investigated retrospectively. The clinical data collected included the age, sex, onset age, clinical symptoms, general head and neck examinations, disease duration, auxiliary examination, diagnosis, treatment, and other clinical information of the children. Auxiliary examinations, including head and neck CT or head and neck MRI, routine blood tests, hepatic function, coagulation, erythrocyte sedimentation rate, CRP, NT-proBNP, ferritin and troponin I, were investigated. The majority of clinical data used in this study were collected from the first day of hospital admission. The CT number of retropharyngeal abnormalities was measured at the site of the central lesion.

### Statistical analysis

All statistical analyses were conducted using SPSS software (version 26.0, SPSS). All statistical significance tests were two-sided, the test level *α* = 0.05, and the difference was statistically significant at *P* < 0.05. The measurement data are expressed as the mean (95% CI), and the enumeration data are expressed as the number of people (%). If the measurement data conformed to a normal distribution, differences between the two groups were compared using a t test; otherwise, the Mann–Whitney U test was used. Differences in the enumeration data between the two groups were compared using the chi-square test.

## Results

### Characterization of study subjects

The demographic information, clinical presentation, auxiliary examination, and treatment of 46 children were reviewed retrospectively. Of these, there were 10 IKD patients initially presenting as retropharyngeal abnormality (Group A), 20 KD patients without retropharyngeal abnormality (Group B) and 16 surgical drainage confirmed retropharyngeal abscess patients (Group C). Data regarding the characteristics of the groups are summarized in Table 1. IKDs initially presenting as retropharyngeal abnormalities were mostly preschool children, and their median age was 65.80 months, with no sexual predilection.

**Table 1:** Characteristic of patients with KD initially presenting as retropharyngeal abnormality and retropharyngeal abscess without KD

Characteristic	Group A (*n* = 10)	Group B (*n* = 20)	Group C (*n* = 16)	*P* value	
				A vs. B	A vs. C
1. Gender					
Male	5 (50.00%)	12 (60.00%)	9 (56.25%)	0.461	0.756
Female	5 (50.00%)	8 (40.00%)	7 (43.75%)		
2. Age (months)	65.80 (50.43–81.17)	31.75 (23.31–40.19)	56.69 (41.61–71.77)	0.000	0.392
3. Clinical symptoms and signs^a^					
Fever	9 (90.00%)	20 (100.00%)	14 (87.50%)	0.333	1.000
Cough	1 (10.00%)	6 (30.00%)	1 (6.25%)	0.372	1.000
Nasal congestion	1 (10.00%)	5 (25.00%)	2 (12.50%)	0.633	1.000
Neck pain	10 (100.00%)	5 (25.00%)	16 (100.00%)	0.000	1.000
Restricted neck movements	10 (100.00%)	5 (25.00%)	15 (93.75%)	0.000	1.000
Pharyngalgia	7 (70.00%)	4 (20.00%)	15 (93.75%)	0.015	0.264
Pain on swallowing	6 (60.00%)	0	11 (68.75%)	0.000	0.692
Pharyngeal congestion	8 (80.00%)	13 (65.00%)	14 (87.50%)	0.675	0.625
Swollen tonsils	6 (60.00%)	2 (10.00%)	13 (81.25%)	0.007	0.369
Lymphadenitis	10 (100.00%)	4 (20.00%)	10 (100.00%)	0.000	1.000
Rash	0	18 (90.00%)	0	0.000	1.000
Nonpurulent conjunctivitis	0	17 (85.00%)	0	0.000	1.000
Cracked lips	0	19 (95.00%)	0	0.000	1.000
Extremities^b^	0	11 (55.00%)	0	0.0004	1.000
Hoarseness	2 (20.00%)	3 (15.00%)	2 (12.50%)	1.000	0.625
4. Fever—highest (°C)	39.76 (39.33–40.19)	39.86 (39.63–40.08)	38.73 (38.20–39.28)	0.643	0.008
5. Fever duration (day)					
Before antibiotic therapy	2.50 (1.37–3.63)	5.00 (3.47–6.53)	4.19 (1.47–6.91)	0.005	0.670
After antibiotic therapy	4.50 (3.42–5.58)	2.10 (1.60–2.60)	1.31 (0.57–2.06)	0.000	0.000
As a whole	7.00 (5.42–8.58)	7.15 (5.72–8.58)	5.43 (2.53–8.34)	0.530	0.071
6. Fever duration after antibiotic therapy (day)					
< 3	1 (10.00%)	9 (45.00%)	13 (81.25%)	0.024	0.001
≥ 3	9 (90.00%)	11 (55.00%)	3 (18.75%)		
7. Laboratory parameters^a^					
WBC (×10^9^/L)	16.35 (12.13–20.56)	14.94 (13.00–16.87)	20.81 (17.31–24.31)	0.567	0.040
Reticulocyte (× 10^12^/L)	4.33 (4.01–4.65)	4.16 (3.99–4.33)	4.39 (4.10–4.68)	0.692	0.768
Haemoglobin (g/L)	116.30 (110.71–121.89)	111.55 (106.91–116.19)	115.81 (109.19–122.43)	0.198	0.913
Platelet (× 10^9^/L)	352.00 (313.98–390.02)	326.65 (263.90–389.40)	411.63 (341.44–481.81)	0.572	0.246
CRP (mg/L)	94.19 (78.68–109.69)	51.89 (34.75–69.02)	58.14 (38.33–77.96)	0.002	0.011
ESR (mm/h)	63.90 (46.35–81.45)	55.20 (48.16–62.24)	65.88 (54.99–76.76)	0.509	0.826
Procalcitonin (ng/mL)	1.25(−0.22–2.72)	2.78(− 0.11–5.67)	0.47 (0.22–0.72)	0.895	0.812
Total protein (g/L)	73.62 (70.72–76.52)	62.89 (60.66–65.11)	74.63 (71.65–77.62)	0.000	0.626
Albumin (g/L)	38.86 (36.72–41.00)	36.05 (34.86–37.23)	38.46 (36.63–40.29)	0.012	0.763
Globulin (g/L)	34.76 (32.62–36.89)	26.84 (24.94–28.74)	35.86 (32.58–39.13)	0.000	0.605
ALT (IU/L)	91.00 (15.04–166.94)	104.00 (50.83–157.17)	13.75 (10.07–17.43)	0.676	0.002
AST (IU/L)	65.60 (28.89–102.31)	70.10 (20.32–119.88)	25.25 (20.50–30.00)	0.692	0.013
Total bilirubin (μmol/L)	14.81 (0.76–30.38)	14.47 (6.10–22.85)	8.18 (4.62–11.93)	0.650	0.623
Direct bilirubin (μmol/L)	9.57 (2.87–22.01)	8.91 (2.70–15.11)	3.66 (2.07–5.26)	0.914	0.262
Alkaline phosphatase (IU/L)	187.60 (153.79–221.41)	203.15 (162.82–243.48)	183.88 (162.83–204.92)	0.843	0.830
γ-glutamyl transpeptidase (IU/L)	46.10 (6.20–86.00)	83.15 (48.60–117.70)	17.56 (12.59–22.53)	0.134	0.427
Na (mmol/L)	134.13 (131.54–136.72)	135.08 (133.87–136.29)	135.39 (133.98–136.80)	0.414	0.317
APTT (S)	41.05 (33.88–48.22)	36.25 (32.68–39.81)	37.13 (32.36–41.89)	0.194	0.309
PT (S)	14.31 (13.48–15.14)	13.69 (13.14–14.24)	13.12 (11.31–14.93)	0.178	0.225
NT-proBNP (ng/L)^d^	247.62 (174.18–321.06)	175.19 (15.91–334.46)	–	0.267	–
Ferritin (μg/L)	489.74 (1.22–980.71)	259.93 (184.15–335.70)	–	0.377	–
Troponin I (×10^−3^ μg/L)	1.9 (0.45–3.35)	4.35 (1.15–9.85)	0.50 (0.06–0.94)	0.681	0.020
8. Radiological findings					
Ring enhancement^c^	0	–	11 (100.00%)	–	0.000
CT Number of RA	21.70 (16.53–26.87)	–	23.50 (17.60–29.40)	–	0.653
9. Hospital length of stay (day)	11.10 (9.37–12.83)	6.70 (5.32–8.08)	8.56 (6.51–10.61)	0.000	0.075

*WBC* white blood cell counts, *CRP* C-reactive protein, *ESR* erythrocyte sedimentation rate, *ALT* alanine aminotransferase, *AST* aspartate aminotransferase, *APTT* activated partial thromboplastin time, *PT* prothrombin time, *INR* international normalized ratio, *FIB* fibrinogen, *RA* retropharyngeal abnormality

^a^Only the clinical symptoms and signs and laboratory parameters of the admission were analysed

^b^Extremities: erythema and oedema of the hands and feet and/or periungual desquamation

^c^Only patients with enhanced imaging were counted

^d^NT-proBNP data were only available for 2 of group A and 4 of group B

### Comparison between group a and group B

The characteristics of patients in Group A and Group B are listed in Table 1. Statistically significant sex differences were not found for the two groups. The age difference was statistically significant (65.80 (50.43–81.17) vs. 31.75 (23.31–40.19); *p* = 0.000), and IKD patients initially presenting as retropharyngeal abnormalities presented more commonly in older children. The fever duration was significantly different between Group A and Group B before antibiotic therapy (2.50 (1.37–3.63) vs. 5.00 (3.47–6.53); *p* = 0.005) and after antibiotic therapy (4.50 (3.42–5.58) vs. 2.10 (1.60–2.60); *p* = 0.000). KD patients without retropharyngeal abnormalities had a longer fever duration before antibiotic therapy and a shorter fever duration after antibiotic therapy. Median CRP (94.19 (78.68–109.69) vs. 51.89 (34.75–69.02); *p* = 0.002) and total protein (73.62 (70.72–76.52) vs. 62.89 (60.66–65.11); *p* = 0.000) were higher in Group A than in Group B patients, but the total protein of most groups was within the normal range.

### Comparison between group a and group C

The characteristics of Group A and Group C are summarized in Table 1. No statistically significant differences were found in demographic data or most clinical manifestations between the two groups, including sex, age, and major clinical symptoms and signs. The patients in both groups mainly presented with fever, neck pain and restricted neck movements on admission The rate of hoarseness in the group A and group C was 20.00 and 12.50%, respectively. However, there was no significant group difference, which was probably related to the small sample size. The fever duration was not significantly different between the two groups. However, there was a significantly shorter fever duration before antibiotic therapy (2.50 (1.37–3.63) vs. 4.19 (1.47–6.91), *p* = 0.005) and longer fever duration after antibiotic therapy (4.50 (3.42–5.58) vs. 1.31 (0.57–2.06), *p* = 0.000) in the KD children initially presenting with retropharyngeal abnormalities. When compared with retropharyngeal abscesses, significantly more children in the KD group experienced fever lasting ≥3 days after antibiotic therapy (9 (90.00%) vs. 3 (18.75%), *p* = 0.001).

In laboratory findings, KD initially presenting as retropharyngeal abnormality had lower WBC (16.35(12.13–20.56) vs. 20.81(17.31–24.31), *p* = 0.040) and higher CRP levels (94.19(78.68–109.69) vs. 58.14(38.33–77.96), *p* = 0.011) than retropharyngeal abscess controls, and the differences were statistically significant. They had significantly higher levels of ALT (91.00 (15.04–166.94) vs. 13.75 (10.07–17.43), *p* = 0.002) and AST (65.60 (28.89–102.31) vs. 25.25 (20.50–30.00), *p* = 0.013). The total and direct bilirubin levels of group A were higher than group C, they may have clinical relevance for people with IKD, but not statistically significant, probably related to small sample sizes. The troponin I level is significantly higher in the group A when compared to group C, but it was below normal and the difference is not large enough to be of clinical relevance. The troponin I level is significantly higher in the group A when compared to group C, but it was below normal and the difference is not large enough to be of clinical relevance. Other laboratory test results (median level of platelets, ESR, Na, and coagulation function) did not differ between the two groups.

**Fig. 1:**
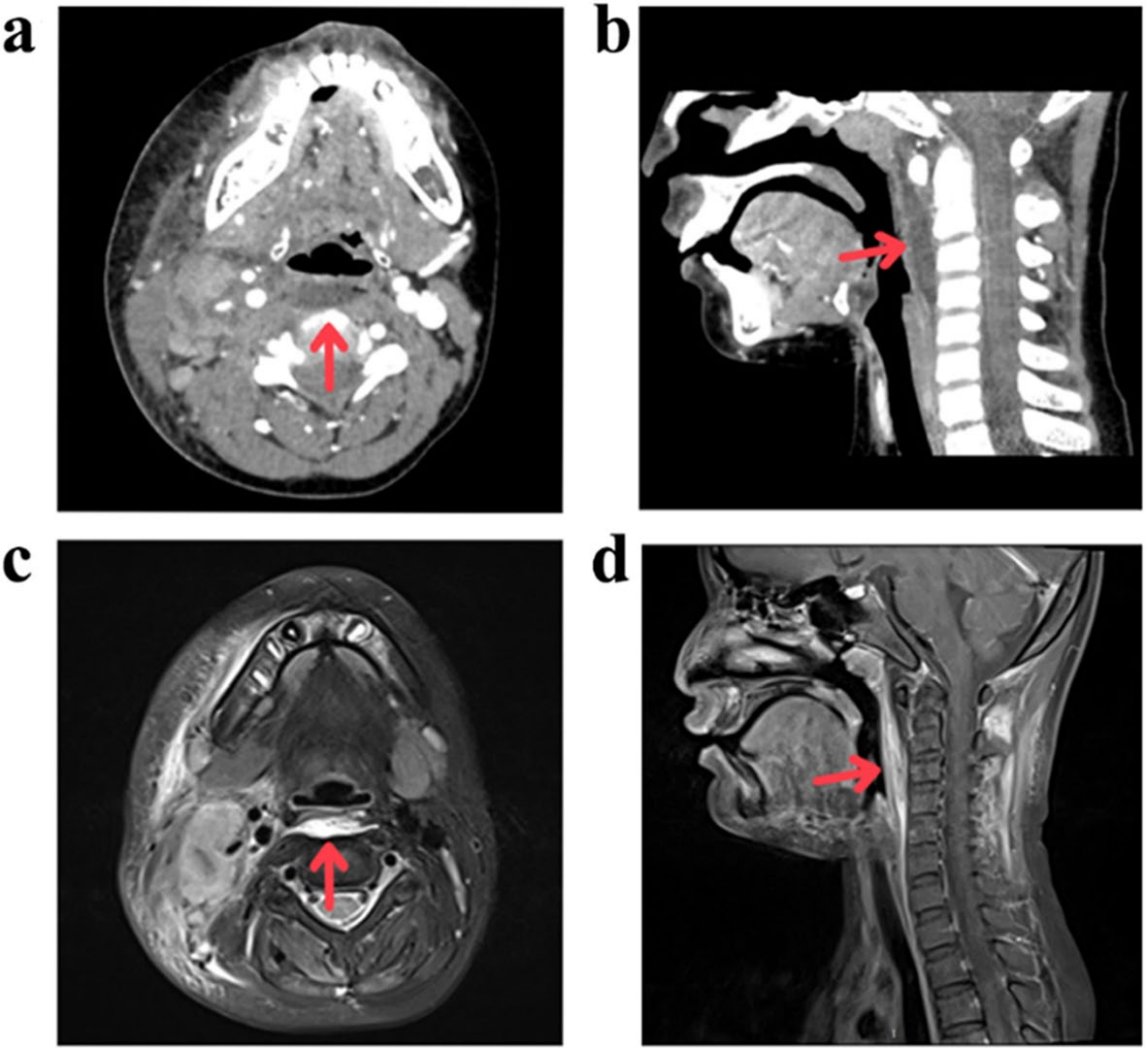
Neck radiological findings of KD initially presenting as retropharyngeal abnormality. **a**, **b** Axial and sagittal enhanced neck CT images showing a widening of the retropharyngeal space with a hypodense area without significant ring enhancement (arrow). **c**, **d** Axial T2fs MR image and sagittal postGd T1fs MR image showing a retropharyngeal fluid collection with soft tissue oedema without significant ring enhancement (arrow)

**Fig. 2:**
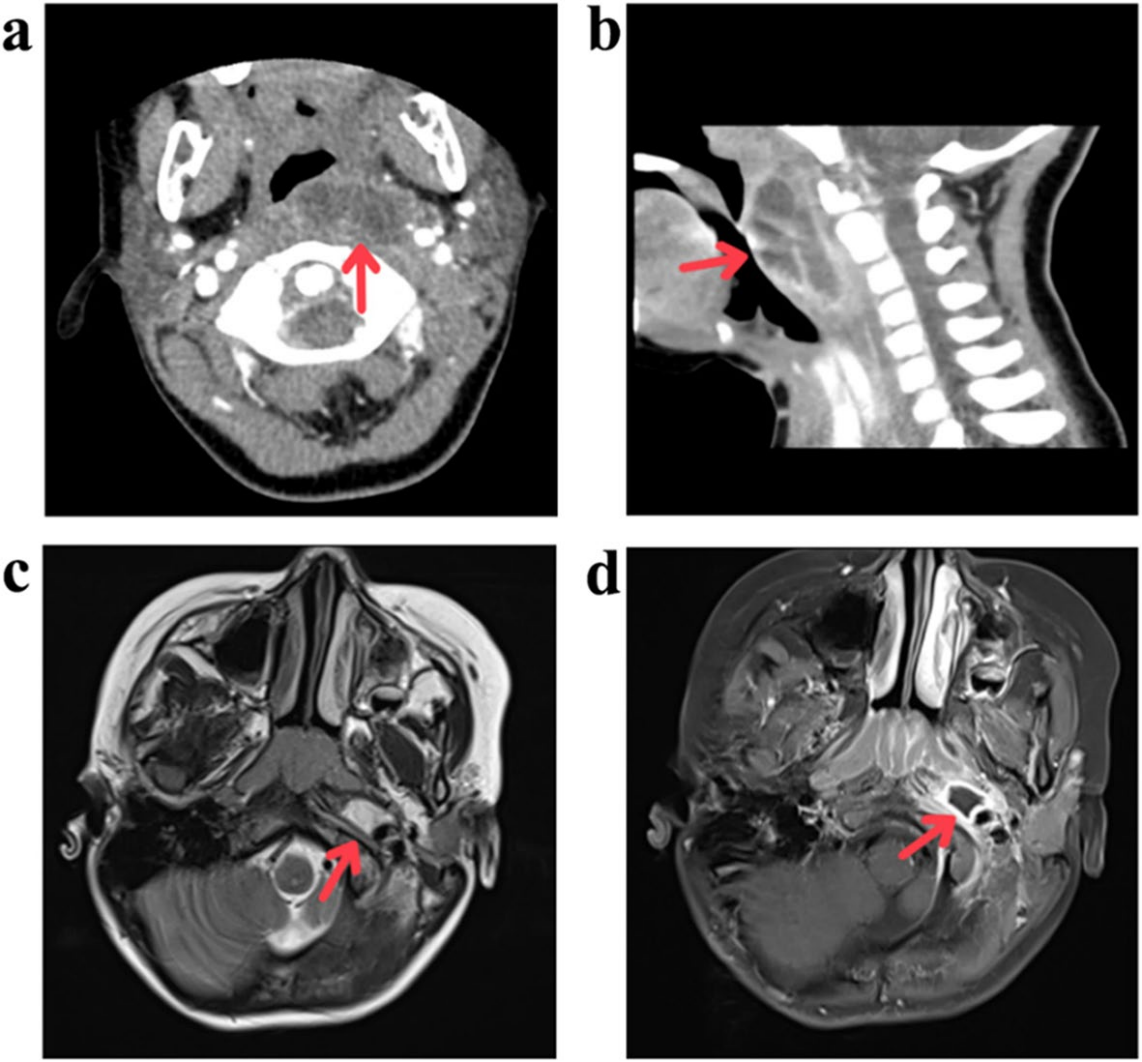
Neck radiological findings of retropharyngeal abscess without KD. **a**, **b** Axial and sagittal enhanced neck CT images showing a large collection filling the retropharyngeal space. There is a marked mass effect and ring enhancement. **c**, **d** Axial T2fs MR image and axial postGd T1fs MR image showing a left-sided retropharyngeal fluid collection with soft tissue oedema with significant ring enhancement (arrow)

In the present study population, 4 patients in Group A and 11 patients in Group C had adequate imaging for enhanced imaging analysis. On all enhanced neck CT scans or contrast-enhanced MR images performed, a retropharyngeal abnormality was found without any peripheral contrast in IKD patients (Fig. 1). Radiological findings revealed ring enhancement in a greater proportion of retropharyngeal abscess without KD patients (*p* = 0.000, Fig. 2). In addition, mass effect is more common in retropharyngeal abscess patients (Figs. 1 and 2). The CT number of retropharyngeal abnormalities did not differ between the two groups.

### Treatment and outcomes

Cases of KD initially presenting as retropharyngeal abnormality are reported in Table 2. All patients initially presented with fever and neck swelling without the additional clinical criteria for the diagnosis of classic KD. The initial clinical presentation was concerning for possible deep neck bacterial infection, prompting us to order imaging studies and initiate antibiotic therapy. They had delayed diagnosis because their conditions were misdiagnosed as retropharyngeal abscess and they underwent antibiotic, corticosteroids or surgical drainage treatment in the early phase of the disease. They all had a persistent fever unresponsive to antibiotics. Antibiotic therapy was escalated to suit 3 patients’ conditions. Surgical drainage was performed on 1 child, but no purulent discharge was obtained after incision, and no microorganisms were cultured. The rash of 4 patients, when it appeared secondarily, was mistaken for an allergic reaction to antibiotics. An accurate diagnosis was made in these cases when fever persisted and other characteristic features of KD eventually developed. The mean time between the onset of illness and KD diagnosis was 6.80 days. After establishing the diagnosis, all patients received high-dose intravenous immunoglobulin (IVIG) and aspirin treatment followed by rapid clinical improvement. Such delays in diagnosis and appropriate treatment led to 2 patients (20.00%) demonstrating abnormal findings on echocardiography (coronary artery dilatation) during the acute illness. All patients were discharged with partial or complete resolution of clinical symptoms and abnormal laboratory findings. After discharge, they continued their treatment with aspirin until complete recovery. The follow-up was conducted 3 months after discharge from hospital, and they all were well with no sequelae and no cardiac involvement.

**Table 2:** Cases of KD initially presenting as retropharyngeal abnormality

Case	Age (m)	Sex	Time from onset to KD diagnosis (days)	KD criteria (day that symptom appeared)	Echocardiogram for coronary artery during the acute illness	Management before diagnosis
1	70	F	4	Ocular (4); Oral (4); Rash (4); Extremities (5)	Normal	Antibiotic + Corticosteroids (parenteral dexamethasone; 0.2 mg/kg/d; 0.4 mg/kg)
2	70	F	6	Ocular (6); Oral (6); Extremities (6)	Dilation of RCA	Antibiotic + Corticosteroids (parenteral methylprednisolone; 1 mg/kg/d; 1 mg/kg)
3	57	F	7	Rash (5); Ocular (7); Oral (7)	Normal	Antibiotic + Corticosteroids (parenteral methylprednisolone; 1 mg/kg/d; 2 mg/kg)
4	90	F	10	Rash (7); Oral (9); Ocular (10); Extremities (10)	Normal	Antibiotic + Corticosteroids (parenteral methylprednisolone; 1 mg/kg/d; 2 mg/kg)
5	33	M	6	Ocular (6); Oral (6); Extremities (6)	Normal	Antibiotic
6	66	M	7	Oral (6); Ocular (7); Rash (7)	Normal	Antibiotic + Corticosteroids (parenteral dexamethasone; 0.2 mg/kg/d; 0.4 mg/kg)
7	52	M	7	Rash (4); Ocular (7); Oral (7)	Normal	Antibiotic + Corticosteroids
8	54	M	4	Ocular (4); Oral (4); Rash (4); Extremities (6)	Dilation of RCA	Antibiotic + Corticosteroids (parenteral dexamethasone; 0.2 mg/kg/d; 0.2 mg/kg)
9	56	M	9	Rash (8); Ocular (9); Oral (9)	Normal	Antibiotic + Corticosteroids
10	110	M	8	Ocular (8); Oral (8); Extremities (9)	Normal	Antibiotic + Corticosteroids (parenteral dexamethasone; 0.2 mg/kg/d; 0.6 mg/kg) + Drainage surgery

*F* female, *M* male, *RCA* right coronary artery, Corticosteroids are expressed as corticosteroids (type; daily dose; accumulative dose)

All typical KD patients without retropharyngeal abnormalities received high-dose intravenous immunoglobulin (IVIG) and aspirin treatment followed by rapid clinical improvement. Only 1 patient (5.00%) demonstrated abnormal findings on echocardiography (coronary artery dilatation) during the acute illness and no cardiac involvement in the follow-up phase.

In the retropharyngeal abscess without KD group, all children underwent surgical drainage and antibiotic therapy. Purulent secretions were seen after incision, and pus culture was positive. All patients were discharged with partial or complete resolution of clinical symptoms and abnormal laboratory findings. No recurrence was observed during the follow-up period of 3 months.

## Discussion

Kawasaki disease is an acute febrile systemic vasculitis of unknown aetiology, and its incidence is increasing yearly [13, 14]. As the aetiology of KD is unknown and there is a lack of specific laboratory indicators for KD, diagnosis still depends primarily on clinical manifestations. Fever, rash, hyperaemia in the bulbar conjunctiva, changes in the lip and oral cavity, peripheral extremity changes, and cervical lymphadenopathy are the main clinical presentations in KD patients. It is not difficult to diagnose KDs when they have a typical clinical manifestation. However, the incidence of IKD has been increasing annually in recent years [15–17]. IKD has fewer characteristic clinical features than KD, causing delays in diagnosis, missed diagnosis or misdiagnosis. Some IKD cases initially presented as only fever and cervical lymphadenopathy and accompanying retropharyngeal low-density areas on CT, mimicking deep neck infection [7, 8, 18]. These cases are frequently misdiagnosed as retropharyngeal abscesss due to atypical symptomatology. However, the treatments of the two diseases are dramatically different, and some cases have been diagnosed when peripheral desquamation occurs in the recovery period, so the incidence of coronary artery disease (CAD) in them is high, which is related to delays in diagnosis [9, 18, 19]. To the best of our knowledge, this is the first retrospective case–control study that has compared the clinical characteristics of IKD patients initially presenting with retropharyngeal abnormalities to both KD patients without retropharyngeal abnormalities and retropharyngeal abscess patients.

Our IKD cases initially appeared both clinically and radiographically as a retropharyngeal space infectious process, and it was not until the other classic clinical manifestations of KD were noted that the correct diagnosis of KD was established. In our study, the incidence of IKD initially presenting as retropharyngeal abnormality in patients with KD was 0.66%; however, the exact incidence has not been defined because cervical imaging studies are not a routine examination for diagnosing KD, and some cases may be misdiagnosed as deep neck infection and resolved with antibiotic and corticosteroid treatment. Tona et al. [3] retrospectively reviewed 277 KD patients and found that low-density lesions in the retropharyngeal space were identified by contrast-enhanced CT in 3.6% of KD patients. A US report with population-based retrospective analysis of serial cross-sectional datasets using the Kids’ Inpatient Database described 20,787 patients with Kawasaki disease, of whom 0.6% (130 cases) had deep neck space involvement, which was similar to our study. However, they included some patients with a previous diagnosis of KD who had deep neck space involvement. The incidence of IKD initially presenting as retropharyngeal abnormality in patients who were suspected to have retropharyngeal infection was 7.52% in our study. Lim et al. [20] retrospectively reviewed the medical records of children diagnosed with parapharyngeal and retropharyngeal cellulitis or abscess using CT at a single institution over a 3-year period. They found that 11 (23.4%) patients were eventually diagnosed with KD. Therefore, if a patient has fever, lymphadenopathy, and oedema of the retropharyngeal space, retropharyngeal infection might be initially suspected, but IKD initially presenting as retropharyngeal abnormality should also be considered.

Although no sex difference was observed in the IKD initially presenting as retropharyngeal abnormality patients, the patients with retropharyngeal low-density lesions were older than those without, suggesting that the more mature mucosal immune system in older patients might result in a more intense inflammatory response. In the early phase of the clinical course, the symptoms of IKD initially presenting as retropharyngeal abnormality include fever, sore throat, neck pain, torticollis or limitation of motion, neck irritation and lymphadenopathy. Some patients had dysphagia, dyspnoea, salivation and hoarseness. Severe cases can lead to wheezing and upper airway obstruction. It is still difficult to distinguish children with IKD initially presenting as retropharyngeal abnormality from children with retropharyngeal abscess in the early phase of the clinical course. On the day of admission, the two diseases had similar early clinical presentations, and there were no significant differences in any major signs or symptoms between the two groups. The rate of hoarseness in the group A, group B and group C was 20.00, 15.00 and 12.50%, respectively. However, there was no significant group difference, which was probably related to the small sample size. Previous studies revealed that hoarseness was found to be prevalent as a presenting sign of acute KD in younger children, it might be related to viral infection and immunological diseases, and hoarseness was presented in 11.6–30.0% patients [21, 22]. However, in children with retropharyngeal abscess, hoarseness often be caused by compression of nerves by abscesses or inflammatory edema in the glottic region, the incidence of hoarseness was relatively low [23, 24]. This symptom might be an important diagnostic clue of KD, but it needs to be confirmed by further large sample studies. However, after admission, fever subsided within 3 days of antibiotic therapy in most children with retropharyngeal abscess, while fever persisted for 3 days or longer in most children with IKD initially presenting as retropharyngeal abnormality despite antibiotic therapy. However, the number of major KD clinical symptoms gradually appeared over time as the course progressed, implying the importance of close observation for the development of new KD signs and symptoms.

In addition, the patients could be distinguished by careful in-depth analysis of laboratory parameters and neck radiological findings. Our study shows that AST, ALT and CRP levels were significantly higher and WBC levels were significantly lower in IKD initially presenting as retropharyngeal abnormalities than in retropharyngeal abscesses. This may be because IKD is a systemic disease and might involve liver injury. ALT and AST are indices of liver function damage. The exact etiology of abnormalities of liver function tests in IKD has not been established. Hypotheses included generalized inflammation, vasculitis, congestive heart failure secondary to myocarditis, nonsteroidal anti-inflammatory antipyretics, toxin-mediated effects, or a combination of these events [25]. And retropharyngeal abscess is more a local infection, this does not appear to affect liver function. In some previous reports, these laboratory findings also differed slightly between the two diseases, but the difference did not reach statistical significance [20, 26]. Furthermore, neck radiological findings, such as the presence of ring enhancement and mass effects on the airway, are also useful tools for distinguishing these two diseases. In all of our IKD children, the main finding was retropharyngeal low density without ring enhancement and mass effects on the airway. Roh et al. [27] reported that the majority of the 34 (61%) of 56 patients with retropharyngeal low density were diagnosed with KD, and retropharyngeal low density without rim enhancement was seen in all KD patients. However, whether the CT number of retropharyngeal abnormalities is useful in distinguishing the two diseases remains controversial. Sasaki et al. reported that they could distinguish lesions from true abscesses by measuring their Hounsfield unit values. However, previous studies and the present study found that there was no difference in the CT number of retropharyngeal abnormalities [26, 28]. Among the types of retropharyngeal inflammation, retropharyngeal low density without rim enhancement indicates retropharyngeal cellulitis, and the circumferential rim of enhancement and mass effects are the hallmarks of an abscess [3, 27]. Neck radiological findings are often performed in children with fever and restricted neck movements. However, whether MRI or CT and whether plain or contrast are selected for imaging is dependent on hospital resources and policy. Plain CT seems to be the modality of choice in the majority of hospitals. However, recommended imaging for suspected KD initially presenting as retropharyngeal abnormality includes contrast-enhanced CT or MR of the neck, and unnecessary surgery for drainage can be avoided.

Compared to KD patients without retropharyngeal abnormalities, we observed that IKD patients initially presenting with retropharyngeal abnormalities were older, ranging in age from 33 to 110 months. This finding may suggest retropharyngeal abnormalities that might be related to the age of KD patients. The more mature mucosal immune system in older patients might result in a more intense inflammatory response, which is why they had higher CRP levels.

The retropharyngeal abscess is serious, potentially lifethreatening deep neck infections in children. The incidence has been reported to have 0.22 cases per 10,000 [29]. Retropharyngeal infections are not rare, and they are far more numerous than IKD initially presenting as retropharyngeal abnormality [29, 30]. Therefore, physicians shouldn’t hesitate to use antibiotics at presentation as retropharyngeal abscess can be fatal. The initial misdiagnosis could be explained by the onset of the disease when the clinical picture was still incomplete. However, the fact that successive antibiotic treatment may not be effective was often interpreted as inefficiencies of treatments. A therapeutic escalation rather than a questioning of the diagnosis could be responsible for a delayed diagnosis. In addition, the rash, when it appeared secondarily, may sometimes be interpreted as an allergic reaction to antibiotics. Corticosteroids were associated with lower odds of surgical drainage among children with retropharyngeal abscess [31], so some children received corticosteroid treatment, which may mask the symptoms of KD and then delay diagnosis. In caring for children with suspected retropharyngeal abscess, particularly when they are not responding to antibiotics, clinicians should evaluate them for the possibility of KD. Fever duration after antibiotics treatment is significantly shorter in retropharyngeal abscess as it indicates the good response to antibiotics in real infection, and not good response to antibiotics should be an alter to look for other etiology including KD.

Currently, the specific mechanisms of KD-induced retropharyngeal abnormalities remain unclear. However, the clinical, operative details, and retropharyngeal abnormality of all cases disappeared following immunoglobulin treatment rather than antibiotic therapy, suggesting that inflammation and oedema were likely the main mechanism, consistent with previously reported literature [8, 9, 18]. In our reported cases and previous cases, surgical exploration of the retropharyngeal area did not reveal areas of fluctuance or abscess, and cultures of the inflammatory exudate were sterile [3–5, 32]. Additionally, it is important to note that there are no reported cases of KD with coexisting retropharyngeal abscess. The retropharyngeal space is anterior to the prevertebral muscles and lies posterior to the pharynx and oesophagus [33]. This space extends from the skull base to the superior mediastinum (the T1-T6 vertebrae) [34, 35]. There is loose connective tissue and abundant lymph nodes within the retropharyngeal space. Lymphadenopathy can lead to lymphatic circulation disorders, accumulation of lymph in the posterior pharyngeal wall, formation of hypodense oedematous areas, and imaging findings similar to posterior pharyngeal abscess, which could explain why the retropharyngeal low-attenuation area seen in neck radiological findings is its extension without significant ring enhancement and mass effects on the airway. The inflammatory responses in the mucosal immune system produce excess levels of inflammatory cytokines during the acute phase of KD [35, 36]. Retropharyngeal low-density lesions in IKD might be caused by a similar mechanism, which could explain why they are seen in older patients whose mucosal immune systems are more mature. Furthermore, previous studies have reported that the retropharyngeal abnormality is likely to be associated with hypoalbuminemia and hyponatremia, but no visible difference was detected between Group A and Group B in our study.

The present study has some limitations, including a small sample size, single-centre design and retrospective design. The location of the study may affect the results of the study as well because KD is more common in the northeastern Asian population [36]. These facts may restrict the generalizability of the present findings.

## Conclusions

Although IKD initially presenting as retropharyngeal abnormality is rare, the careful observation of the signs and symptoms of this disease and the comprehensive analysis of the laboratory tests and the neck radiological findings could detect the disease early and then reduce the unnecessary use of antibiotics, reduce unnecessary invasive procedures such as surgical aspiration, and avoid the occurrence of serious complications. The possibility of KD should be considered in children showing deep neck inflammation unresponsive to empirical antibiotic therapy after 3 days and low-density cervical lesions demonstrating minimal to no enhancement. When children initially present with retropharyngeal abnormalities, paediatricians, otolaryngologists, and radiologists should be aware of it as an atypical presentation of KD.

## Data Availability

The datasets used and/or analysed during the current study are available from the corresponding author (YS.T, tys118@163.com) on reasonable request.

## Abbreviations

KD: Kawasaki disease
CAAs: coronary artery aneurysms
IKD: Incomplete Kawasaki disease
CRP: C-reactive protein
WBC: White blood cell counts
ESR: Erythrocyte sedimentation rate
ALT: Alanine aminotransferase
AST: Aspartate aminotransferase
APTT: Activated partial thromboplastin time
PT: Prothrombin time
INR: International normalized ratio
FIB: Fibrinogen
RA: Retropharyngeal abnormality
CAD: Coronary artery disease
CI: confidenceinterval
